# Thermal inkjet makes label-free single-cell proteomics accessible and easy

**DOI:** 10.3389/fchem.2024.1428547

**Published:** 2024-08-21

**Authors:** Stanislau Stanisheuski, Arpa Ebrahimi, Kavi Aashish Vaidya, Hyo Sang Jang, Liping Yang, Alex Jordan Eddins, Carrie Marean-Reardon, Maria Clara Franco, Claudia Susanne Maier

**Affiliations:** ^1^ Department of Chemistry, Oregon State University, Corvallis, OR, United States; ^2^ Department of Biochemistry and Biophysics, College of Science, Oregon State University, Corvallis, OR, United States; ^3^ HP Inc., Corvallis, OR, United States; ^4^ Center for Translational Science, Florida International University, Port St. Lucie, FL, United States; ^5^ Department of Cellular and Molecular Medicine, Herbert Wertheim College of Medicine, Florida International University, Miami, FL, United States

**Keywords:** single-cell proteomics, thermal inkjet, single-cell dispensing, label-free, automation, accessible, commercially available

## Abstract

In this study, we adapted an HP D100 Single Cell Dispenser – a novel low-cost thermal inkjet (TIJ) platform with impedance-based single cell detection – for dispensing of individual cells and one-pot sample preparation. We repeatedly achieved label-free identification of up to 1,300 proteins from a single cell in a single run using an Orbitrap Fusion Lumos Mass Spectrometer coupled to either an Acquity UPLC M-class system or a Vanquish Neo UHPLC system. The developed sample processing workflow is highly reproducible, robust, and applicable to standardized 384- and 1536-well microplates, as well as glass LC vials. We demonstrate the applicability of the method for proteomics of single cells from multiple cell lines, mixed cell suspensions, and glioblastoma tumor spheroids. As additional proof of robustness, we monitored the results of genetic manipulations and the expression of engineered proteins in individual cells. Our cost-effective and robust single-cell proteomics workflow can be transferred to other labs interested in studying cells at the individual cell level.

## 1 Introduction

The majority of applied proteomics studies are performed with heterogeneous cell populations, which results in the loss of protein content information for individual cells. Analyzing cells one at a time eliminates this diluting effect, but proteomic analysis of single cells becomes more challenging due to low protein levels available for sample preparation and mass spectrometric analysis.

Recent advances in mass spectrometry technology and bioinformatics made proteomic analyses of low numbers of cells and single cells possible. At present, it is reasonable to expect a label-free identification of 800–2000 proteins from single cells per run. Depending on the number of samples and whether or not a library was used, data-independent acquisition strategies and match-between-runs (MBR) algorithms can boost the number of IDs achievable in a single run by 30%–80% (Cong et al., 2021; Brunner et al., 2022; Matzinger et al., 2023a).

There is an emerging need for robust technologies that enable rapid isolation of single, viable cells without perturbations to cellular homeostasis. The most common techniques currently applied to single cell proteomic analyses are (1) fluorescence-assisted cell sorting (FACS) and (2) manual micromanipulations (Hu et al., 2016; Matzinger et al., 2023a). Automated single cell dispensing systems have become available commercially and now are suitable for applications in common laboratory settings. Piezoelectric inkjet technology had long been the most prominent alternative to FACS in the field of automated single cell handling (Ctortecka et al., 2021; Vallone et al., 2020; Jagnandan and Morachis, 2022) but an increasing number of new technologies are being adapted for single cell manipulation and selection (Elitas et al., 2014; Takagi et al., 2019; Rienzo et al., 2021; Matsumoto et al., 2022; Radfar et al., 2022).

Having the ability to manipulate single cells is insufficient to perform single cell mass spectrometry. It is a combination of miniaturization, reliable dispensing of reagents, and automation that enables reproducible lossless sample preparation workflows, which is a prerequisite for label-free single-cell proteomics (Slavov, 2021). Given that single mammalian cells contain on average a few hundred picograms (pg) of total protein, the exclusion of error-prone manual sample handling steps and downscaling of sample size is vital to the success of the analysis.

Over the last decade, multiple generations of mass spectrometers with picogram protein sensitivities have become available. Concomitant advancements in chromatographic separation technology and column manufacturing have increased peak capacity and suitability for the separation of minute amounts of analytes. However, the high cost and complexity of robotic fluidic handling have limited the access of less specialized laboratories to single cell proteomic workflows and adaptation (Slavov, 2022).

In addition to automation and miniaturization, another important aspect of single cell handling is a single cell detection system. FACS relies on light scattering for detection and sorting. Other techniques rely on high-content imaging and image processing for the detection of single cells. Novel single cell detection systems based on electrical impedance spectroscopy have been reported as well. For example, impedance-based detection is widely used in Coulter counters and is known for its rapid processivity. Recent advancements in this technology made it compatible with microfluidics and single cell dispensing including providing information about cell size and shape (Cottet and Caselli, 2022; Hannart et al., 2022).

Here, we describe the evaluation and adaptation of a novel low-cost thermal inkjet platform (TIJ) with impedance-based detection for dispensing of individual cells and automated sample preparation for proteomics of individual mammalian cells. TIJ has its origin in the inkjet printing technology used in regular ink printers and has been successfully used in mammalian cell printing for tissue engineering (Cui et al., 2012). We show that the tested TIJ platform with single-cell sensing technology allows rapid dispensing of individual cells, while preserving cell viability, and can be conveniently integrated into proteomics sample processing workflows.

Due to the previously demonstrated ability to accurately dispense exceptionally low liquid volumes (down to 10 pL) (Jones et al., 2013), TIJ can reduce total sample preparation volumes to low nanoliters. We have developed a one-pot sample preparation workflow that is compatible with commercially available microplates and glass LC vials and, thus, fully compatible with major LC autosamplers. The workflow was applied for obtaining single cell proteomic samples from five mammalian cell lines grown in culture, HEK293A, HEK293/eGFP (enhanced green fluorescent protein), HEK293/sfGFP (super-folder GFP), MDA-MB-231, and A549. We also demonstrate detection and quantification of engineered Green Fluorescent Proteins in transiently and stably transfected single HEK293 cells. Stable GFP-transfected cells were also used for evaluating cell viability and demonstrating fitness for single-cell cloning.

In sum, we introduce here a robust TIJ-enabled label-free single-cell proteomic workflows that can be widely adopted by the research community due to its affordability and utilization of components that are commonly available.

## 2 Methods

Brief descriptions are provided below; more experimental details are available in the supporting information.

### 2.1 Cell cultures

HEK293A, A549, and MDA-MB-231 cell lines were purchased from ATCC. HEK-293/GFP stable cells (HEK293/eGFP) were purchased from GenTarget. Custom super-folder GFP producing HEK-293T cells (HEK293/sfGFP) and pAcBac1-sfGFP plasmid were a courtesy of the Ryan Mehl laboratory, Oregon State University.

Mammalian cells were cultured in the presence of 5% CO_2_ at 37°C in complete cell culture media: Dulbecco’s Modified Eagle’s Medium with 4.5 g/L glucose and L-glutamine, without sodium pyruvate (Corning) supplemented with 10% FBS (ScienceCell) and 1% penicillin-streptomycin (Corning). Cells were sub-cultured upon reaching 75% confluency.

After rinsing the cells with Dulbecco’s phosphate-buffered saline DPBS (Corning) followed by incubation with trypsin (0.05% Trypsin-EDTA, Corning) for 3 min, trypsin was neutralized by mixing the cell suspension with complete cell culture media. The cell suspension was gently spun down at 360 × g, the residual growth media were removed, and cells were washed 2 times by resuspension in phosphate-buffered saline (PBS). Optionally, benzonase was added to prevent cell clumping. The resulting cell suspensions were directly used in single cell dispensing experiments.

### 2.2 Single cell cloning and outgrowth

Cells were dispensed into 96-, 384-, and 1536-well plates prefilled with conditioned growth media taken from growing culture of corresponding cells. TC-treated and CellBind microplates were used in these experiments. Imaging was performed using an Evos M7000 system and Keyence BZ-800 fluorescence microscope. Outgrowth was monitored every 24 h. Different ratios of fresh and conditioned media were tested to optimize outgrowth rates.

### 2.3 Low-cell-count sample preparation

Dispensing of reagents and cells was performed using the HP D100 single cell dispenser. 100, 1,000, and 10,000 cells were dispensed into 384-well plate (CellBind, Corning) prefilled with 10 μL of 5 mM dithiothreitol (DTT) in 50 mM ammonium hydrogen carbonate (AmBiC) (LiChropur, Sigma Aldrich). The cell suspensions were dispensed by volume and the cell count was confirmed using imaging. After incubation at 75°C for 1 h, 10 mM iodoacetamide (IAM) and 1 ng/μL trypsin was added. Samples were incubated for 4 h at 37°C and spun down at 2,000 × g to collect insoluble material at the bottom of the plate. Samples were analyzed using method 3 (Supplementary Table S2).

### 2.4 Generation and harvesting of U87-MG-GFP tumor spheroids

Single-cell suspensions of U87-MG-GFP cells harvested from 2D cell cultures were transferred to low attachment plates (Nunclon™ Sphera™ 90 mm Dish, Thermo Scientific™). The media were replenished every 48 h and tumor spheroid formation was monitored every 48 h using a Keyence BZ-800 fluorescence microscope (Supplementary Figure S1).

After 8 days of growth, the spheroids were detached and dissociated in 0.05% Trypsin-EDTA solution using the same conditions as for all other cell types described above except for the addition of vigorous resuspension by pipetting.

### 2.5 Single cell dispensing and trypsin digestion

**FIGURE 1:**
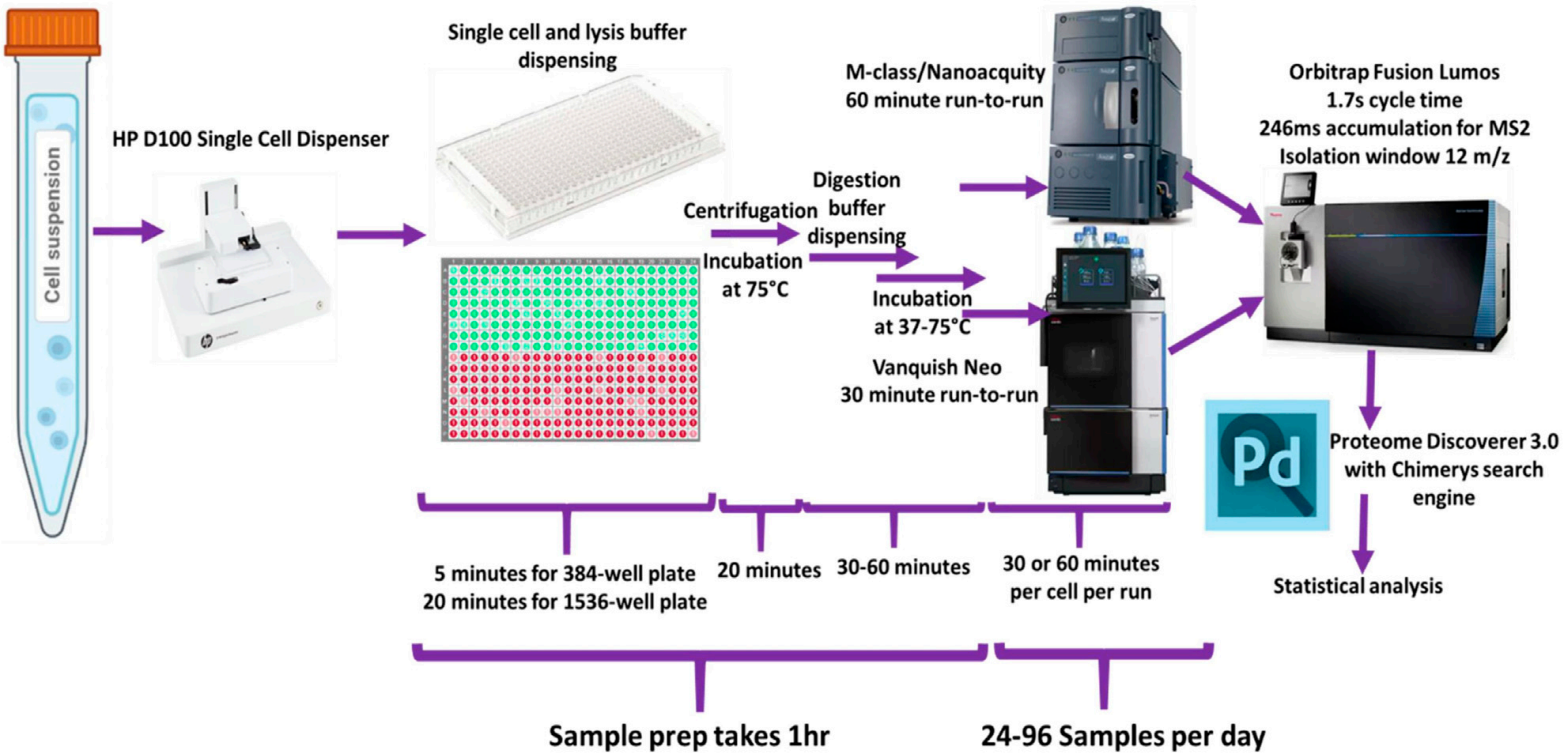
Schematic of overall study design used for the single-cell sample processing workflow and data acquisition.

Dispensing of reagents and cells was performed using the HP D100 single cell dispenser. (Supplementary Table S1). Figure 1 shows a schematic representation of the general workflow.

Before dispensing single cells, plates or vials were prefilled with the 50 mM AmBiC containing required amounts of trypsin and DTT. Cell suspensions, containing 100–1,000 cells/µL, were used for single-cell dispensing. Plates containing cells were centrifuged at 2,000 × g for 5 min and incubated for 10–30 min at 75°C to lyse cells and reduce disulfide bonds, followed by dispensing of digestion buffer (30 mM IAM and 10 ng/μL trypsin in 50 mM AmBiC). Trypsin digestion duration was optimized in the range of 30 min to 4 h and at temperatures from 37°C to 75°C.

Optional steps: 1) MassPREP peptide standard in 50 mM AmBiC was added as an internal standard; 2) 30% acetonitrile was added to facilitate cell lysis; 3) benzonase was added to remove nucleic acids; and 4) 50 mM AmBiC was replaced by 50 mM PBS.

### 2.6 Nano LC-MS setups

Data were generated using a Thermo Fisher Scientific Orbitrap Fusion Lumos connected to a nanoflow UPLC. We evaluated our workflows using the following nanoLC systems: 1) Waters M-class UPLC, 2) NanoAcquity UPLC, and 3) Thermo Vanquish Neo UHPLC (Supplementary Table S4). The following C18 analytical columns were used: 50 μm, 75 μm and 100 µm i.d., with 150 mm column length. For trapping, C18 columns with 75 μm, 180 μm, and 300 µm i.d. were used (SI Appendix 1). Samples were injected from LC vials, 384- and 1536-well plates (more detailed description of consumables used in this study is provided in the Supplementary Material).

Gradient length varied from 2 to 60 min. The LC-MS/MS workflow was optimized for low cell counts using single-cell samples and different concentrations of commercially available tryptic digest of HeLa lysate, as detailed in the Results and Discussion sections. We tested multiple LC setups and analytically validated our optimized workflow, including determining detection limits, dynamic range, reproducibility, and robustness of our single-cell proteomics protocol.

MS data acquisition parameters are listed in Supplementary Table S3.

### 2.7 Data processing and statistical analysis

Raw files for data-dependent acquisition (DDA) mode were processed by Proteome Discoverer 3.1. Sequest HT and Chimerys search engines were used to search against the human reference proteome from Uniprot (Swissprot) database. Selected datasets were reprocessed using MaxQuant 2.4 and Andromeda search engine with the same search parameters. Precursor ion mass tolerance was set to 5 ppm, while fragment ion mass tolerance was 0.02 Da. Raw files for data-independent acquisition (DIA) mode were processed library-free in DIA-NN 1.8.1 FragPipe, and Chimerys. Built-in Proteome Discoverer tools and custom Python and Julia scripts were used for statistical data processing, organizing data, and visualization.

## 3 Results and Discussion

### 3.1 Evaluation of single cell dispensing efficiency and reagent dispensing

We received the HP D100 single cell dispenser as an early prototype. To adapt it for single cell proteomics, it was critical to evaluate its performance in accurate reagent dispensing and dispensing of single and viable cells. During the development phase, software versions and equipment capabilities were continuously updated, adjusted, and benchmarked to enable single cell proteomic workflows. All experiments and data reported here are applicable to the version of the instrument and software commercially available since January 2023 and marketed under the name Tecan Uno Single Cell Dispenser™.

#### 3.1.1 Solution and reagent dispensing

Accurate reagent dispensing is an essential prerequisite for an automated and lossless single-cell proteomics workflow. Thus, we evaluated the precision of reagent dispensing by the D100 system by evaluating the dispensed volume using absorbance and peak areas of peptide ion signal obtained by mass spectrometry (MS1 only). For instance, dispensing of Angiotensin peptide DRVYIHPF (523.7745++) from the MassPrep peptide standard solution down to 20 pL appeared to be very precise showing linearity across at least six orders of magnitude with R^2^ = 0.99 (Supplementary Figure S2).

#### 3.1.2 Single cell dispensing evaluation and viability studies

**FIGURE 2:**
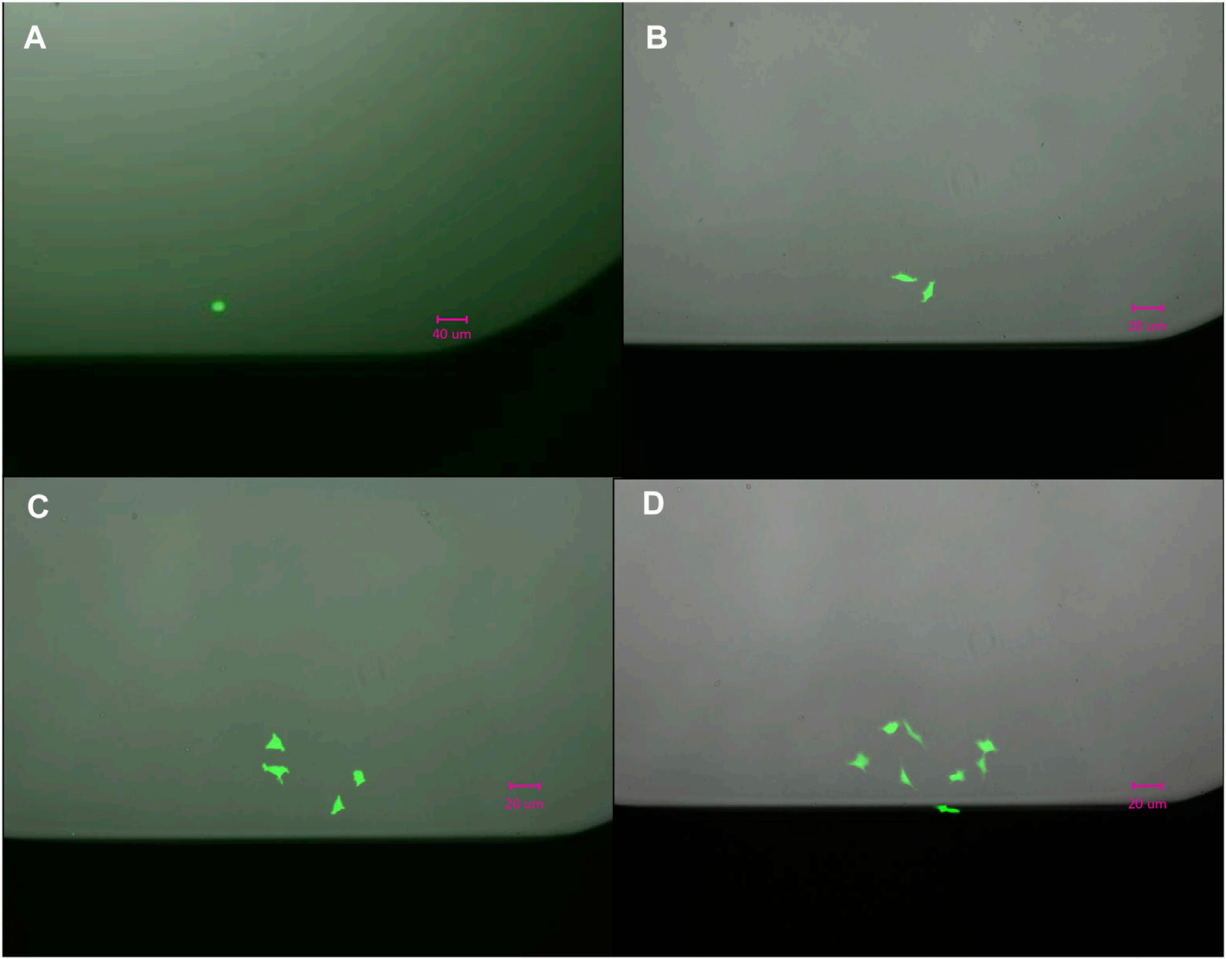
Outgrowth of a single **(A)** HEK293/GFP cell into two in 24 h, **(B)** into four in 48 h **(C)**, and eight **(D)** cells in 72 h.

We evaluated the single cell dispensing performance with respect to dispensing individual cells into wells of commonly used low protein-binding polypropylene and polystyrene microwell plates. The speed of single cell dispensing is high, seeding a full 384-well plate in less than 5 min. For visualizing correct cell placement, we initially used intracellular dyes, such as calcein-AM and trypan blue, and cells transiently expressing GFP. However, these methods rely on dyes or GFP-expressing vectors penetrating the cells and do not reach 100% labeling efficiency. To circumvent this deficiency, we used stably-expressing-GFP HEK293 cells dispensed into 96-, 384-and 1536-well plates to further confirm dispensing of 1 cell per well (Figure 2A). We summarized our findings in Table 1. Approximately 92% of wells of 384- or 1536-well plates reported by the D100 as containing a single cell were confirmed visually to contain single cells. The large-scale manual verification process used to verify single-cell dispensing is prone to errors in cases when the dispensed cells exhibit low fluorescence levels and/or were located on the very edge of the well, thus the efficiency may be higher than the estimated 92%.

**TABLE 1:** Summary of single cell dispensing characteristics.

	Single cells reported by D100	Junk wells reported by D100	Visually confirmed single cells	Single cell reporting efficiency (%)	Fraction of confirmed single cells on the plate (%)
384-well plate 1	353	31	328	92.9	85.4
384-well plate 2	363	21	329	90.6	85.6
384-well plate 3	351	33	324	92.3	84.3
1536-well plate	1,397	139	1,299	92.9	84.6

### 3.2 Cell viability based on GFP expression and cell proliferation after single-cell cloning

It has been previously shown that changes to the cell metabolome can occur in a matter of seconds (Martano et al., 2015; Lu et al., 2017), and to the transcriptome in minutes (Fasching et al., 2022; Overbey et al., 2022); estimates for proteome stability vary, especially when considering posttranslational modifications (Shao et al., 2019; Gegner et al., 2022). It was important to evaluate if the TIJ process and dispensing using the D100 platform affected cell viability, despite that cell viability studies using similar devices were reported previously (Takagi et al., 2019; Yumoto et al., 2020).

Single-cell viability is quite challenging to determine. Conventional viability tests, such as MTT, are inapplicable to single-cell samples. We used the following two markers to confirm single-cell viability: 1) Continuous GFP production as an indicator of cell membrane integrity and proteome homeostasis; and 2) proliferation as an attribute of cell viability.

The images, shown in Figure 2, confirm that dispensed cells continued expressing GFP 24, 48, and 72 h after dispensing and continued dividing over time, indicating that cell was not compromised during dispensing. The outgrowth rate in single-cell cloning experiments in microplates was found to be on average 65% ± 5%, which is comparable to outgrowth rates recently reported using other technologies (Munoz and Morachis, 2022).

### 3.3 Proteome profiles of dispensed and hand-pipetted cells show high consistency

**FIGURE 3:**
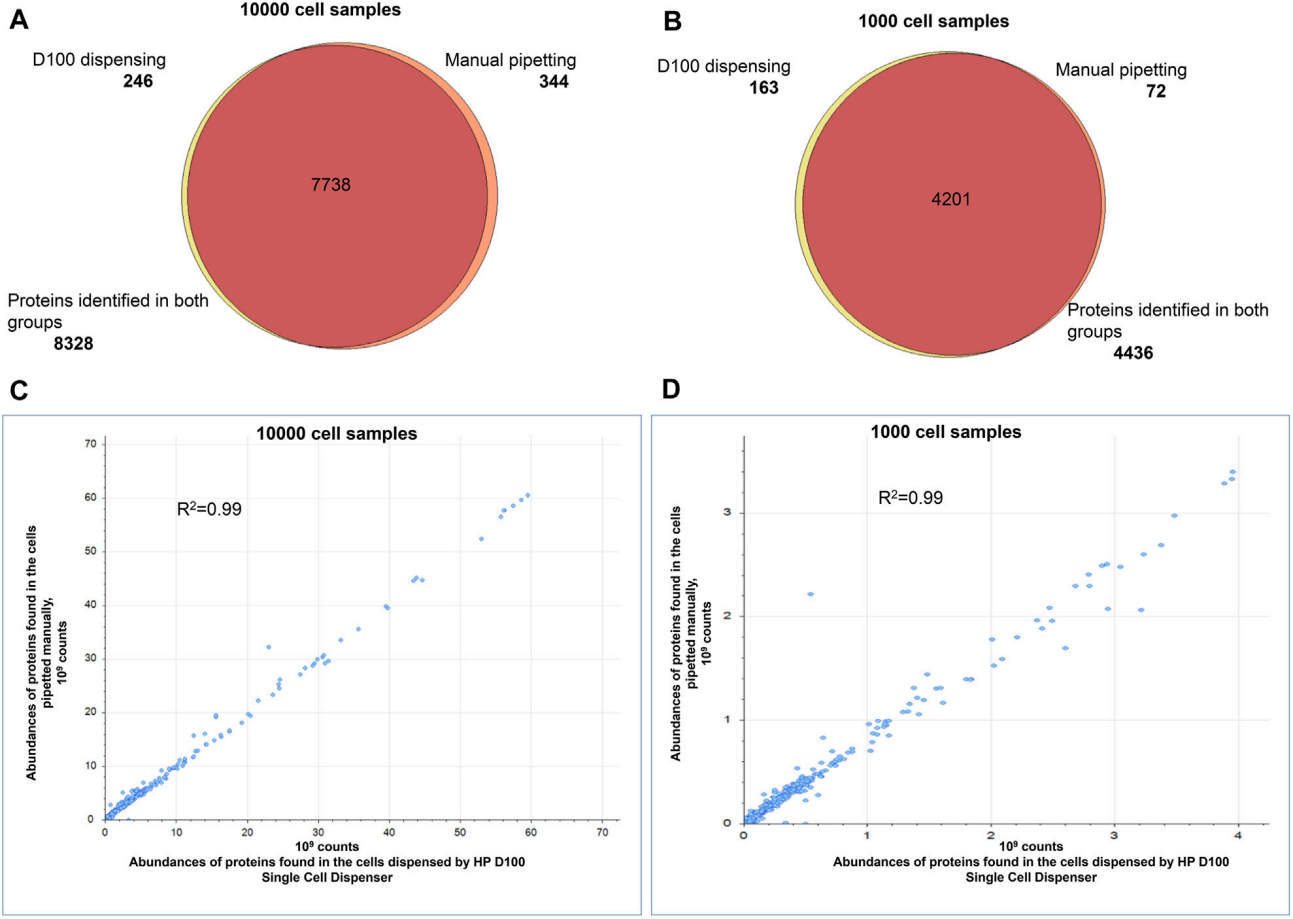
Comparison of the proteomes identified in 1,000 and 10,000 HEK293 cells. Automated dispensing by D100 vs. manual pipetting. Venn diagram comparing the proteins in the tryptic digests of samples prepared by automated dispensing (using the D100) or by transfer by hand-pipetting of **(A)** 10,000 cells, **(B)** 1,000 cells. Pearson’s correlation of protein abundances quantified in the samples prepared by automated dispensing (using the D100) or by transfer by hand pipetting of **(C)** 10,000 cells, **(D)** 1,000 cells. Every sample was prepared and analyzed in quintuplicate.

Next, we compared proteome profiles of cells dispensed by the D100 to cells pipetted by hand to confirm minimal or no impact to the protein content during automated dispensing. We dispensed 100, 1,000, and 10,000 HEK293 cells in bulk or pipetted approximately equal numbers of cells by hand. We acquired proteomic profiles of manually dispensed and ink-jetted cells in quintuplicate. For 10,000 cells we obtained more than 8,300 protein IDs for both groups. The analysis of 1,000 and 100-cell samples resulted in approximately 4,400 and 2,200 proteins, respectively, and found consistently in both groups. There was >95% overlap in proteins identified in both sample groups (Figure 3A). The quantities of identified proteins observed for manually dispensed and ink-jetted cells correlated well, with a Pearson’s coefficient of correlation greater than 0.99 (Figure 3B).

### 3.4 Optimization of single-cell proteomic sample processing workflow including lysis, trypsin digestion conditions and evaluation of protein contaminants

Cell lysis and proteolysis: We assessed existing cell lysis methods used for single-cell proteomics (Ctortecka et al., 2021; Williams et al., 2020; Specht et al., 2021; Budnik et al., 2018; Schoof et al., 2021; Petelski et al., 2021; Specht and Slavov, 2021; Matzinger et al., 2023b). A strong emphasis was placed on lossless and fast methods that prevent evaporation and condensation, and omit the need for humidity control.

**FIGURE 4:**
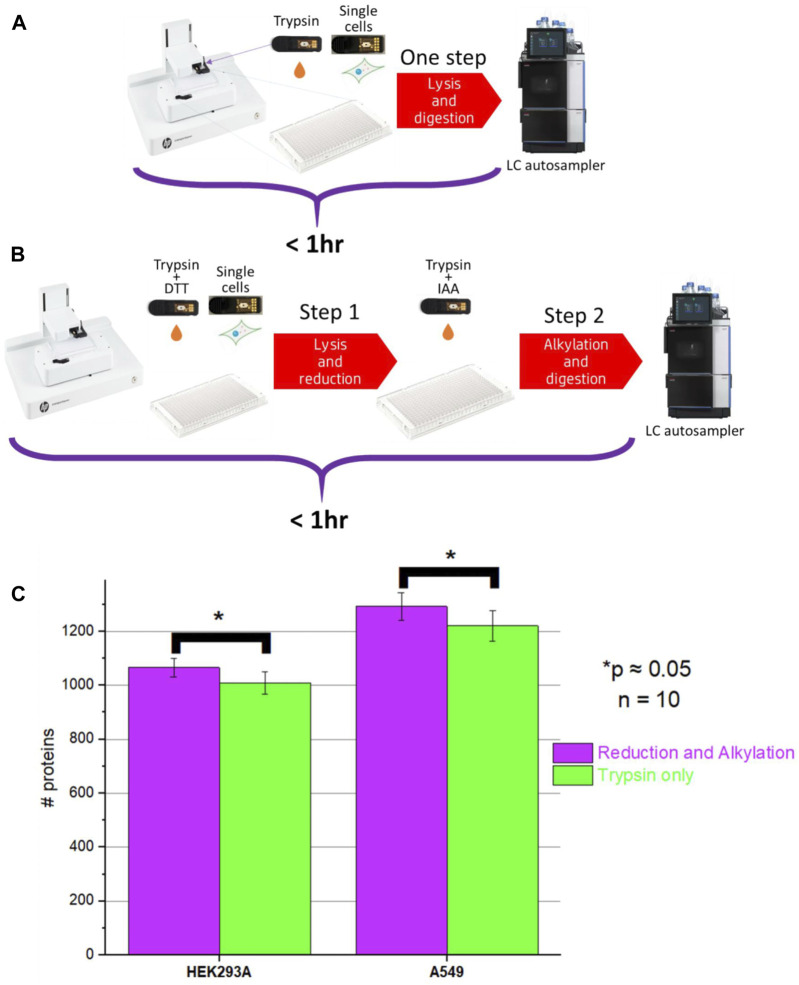
Schematics of **(A)** One-step (no reduction and alkylation) and **(B)** Two-step sample processing (with reduction and alkylation) workflows; **(C)** Comparison of protein ID counts obtained for the one-step and two-step method.

Multiple single-cell sample preparation protocols were explored and after very thorough optimization of sample preparation conditions, we ended up with two sample preparation protocols: two-step and one-step workflow (Figure 4, full description provided in Supplementary Table S1).

We noticed that, unlike in bulk proteomics, due to the very low protein amounts present in the single-cell samples, very high trypsin/LysC digestion efficiency can be achieved with relatively short incubation times even without reduction and alkylation. Additionally, with the elevated incubation temperature and high amount of the proteases used, the incubation time can be shortened even further down to <30 min.

One-step workflows resulted in protein counts comparable to the counts obtained using the two-step workflows. However, there was a minor but statistically significant improvement (*p* = 0.049 and *p* = 0.051 for A549 and HEK293A cells, respectively) (Figure 4) when the two-step workflow was used. Upon closer evaluation, we noticed that the difference was associated with cysteine-containing peptides. It was impossible to evaluate this difference using the Chimerys search engine used for producing Figure 4. Chimerys engine was trained on alkylated samples, so it recognizes exclusively alkylated cysteine-containing peptides and it enforces carbamidomethylation as a static modification. For tracking of cysteine-containing peptides, we used Sequest HT search engine and noticed that the abundance of some cysteine-containing peptides varied the longer the sample remained in the autosampler, whereas the abundance of other peptides remained at the same level. A possible explanation is that without alkylation, cysteine-containing peptides are prone to redox reactions.

It is worth noting that among all peptide sequences identified in single-cell samples, 10%–15% are cysteine-containing sequences. 5%–8% of them result in unique protein identifications, and only a fraction of them degrades over time without alkylation. Many applications do not require the identification and quantification of cysteine-containing peptides, for instance, as described below, the detection of engineered proteins in single cells. For such applications, given comparable proteome coverage, the one-step method would be acceptable and possibly preferable.

After optimizations, the throughput of developed sample preparation workflows reached up to 15,000 single-cell samples per day. In the current work, we focused on the applications important for synthetic biology: we show successful detection of an engineered protein in a single cell and as proof of concept single-cell cloning experiments with stable HEK cells. The ability of the TIJ technology to dispense reliably thousands of intact living single cell will be useful for high-throughput cell line development (Tejwani et al., 2021; Yang et al., 2022) and functional assays at the single cell level (Lalli et al., 2020; Regan and Smalley, 2020; Heumos et al., 2023).

Evaluation of protein-contaminants in single cell proteomic samples: To evaluate the level of possible contaminants we prepared two sets of blanks. “No cell” blanks were prepared by transferring 400 pL of centrifugated for 5 min at 800 × g cell suspension used for the single cell dispensing (500 cells/µL). “Reagent only blanks” were produced by the injection of the reagents without any biological material. “Reagent only blanks” resulted in no protein identifications beside the trypsin and LysC. Notably, “No cell” blank samples resulted in ≈100 protein IDs.

**FIGURE 5:**
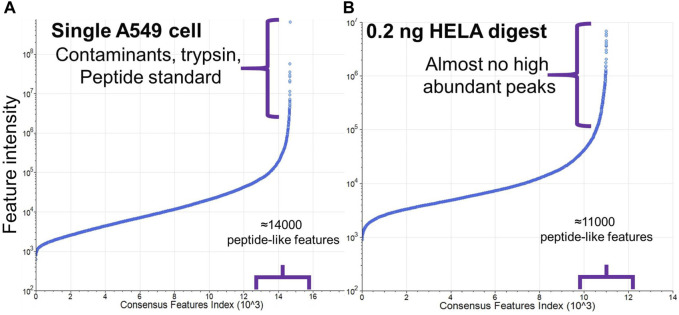
Comparison of abundances of peptide-like features with charge state 2+ and higher detected in **(A)** single A549 cell sample and **(B)** 0.2 ng HeLa digest. *Y*-axis in plot **(A)** was adjusted to include higher ion intensities from the contaminants and trypsin.

Protein contaminants were almost entirely absent when using diluted commercially produced tryptic digests, e.g., HeLa digest, due to the conditions of manufacturing and much lower trypsin-to-protein ratio used for the digestion. However, in the single-cell proteomic samples, small amounts of bovine serum albumin along with other proteins/biomolecules from FBS-containing media attach tightly to the cell surface and some protein-contaminants were detected (Figure 5), even after rinsing the cells multiple times (Frankenfield et al., 2022). Given that extensive rinsing can damage the cells, we performed two rinsing steps in our workflow. As is shown below, the presence of contaminants did not preclude the identification of endogenous proteins. Comparable levels of contaminants have been observed in other workflows as well (Matzinger et al., 2023b). Due to the nature of the single-use cassette system, the possibility of carryover or cross contamination is excluded.

**FIGURE 6:**
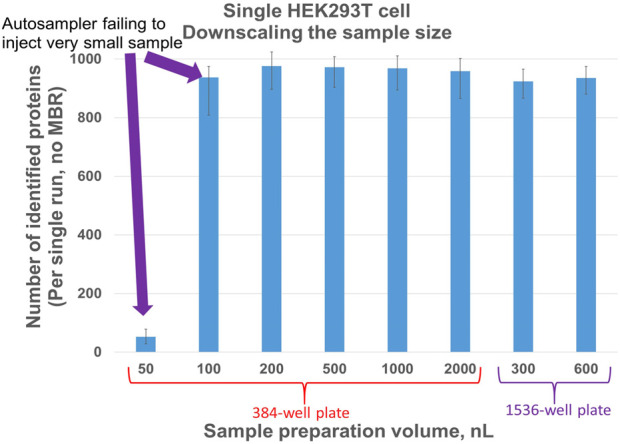
Number of proteins identified for different volumes of single HEK293T cell sample preparation in 384- and 1536-well plates. Injection volume equals 95% of sample preparation volume. Each column represents four single-cell samples.

Evaluation of sample preparation volume to optimize number of protein IDs in single cell samples: Prior works showed that decreasing sample volume is beneficial for proteome coverage (Williams et al., 2020; Cong et al., 2021; Ctortecka et al., 2021). As of to date, none of the commercially available nanoLC autosamplers can perform an injection of only a few nanoliters. With sample volumes that low, evaporation becomes a critical problem, but to a lesser extent in 1536-well plates. Additionally, no significant differences were observed in the sample preparation volume range of 200–2000 nL (Figure 6) if the same total amount of trypsin was used and the injection volume was 95% of the sample volume.

To investigate the proteome coverage for subcellular protein amounts, we increased the volume of a single cell preparation to 4 μL and performed three 1 μL injections for each sample. Injections performed from the same vial were very reproducible. Every injection resulted in the identification of on average 532 proteins for seven single HEK293 cells, and 518 proteins for seven single MDA-MB-231 cells. After applying the MBR algorithm to the whole dataset containing 14 cells (three injections per cell), 1,083 proteins were identified in both cell lines. Even such a small dataset was sufficient to separate the 2 cell types based on their proteome profile using principal component analysis (PCA) (Supplementary Figure S3).

### 3.5 Optimization of the LC-MS data acquisition method

Robust label free single cell proteomics workflows require a thorough optimization of LC gradient and acquisition parameters. Intensity threshold for triggering MS2 event should be low enough to get the fragmentation spectra for as many peptides and high enough to cut out noise signals. All methods described below were developed and tested extensively on the commercially available standard of tryptic HeLa digest diluted to concentrations from 100 pg/μL to 2 ng/μL and on single-cell samples.

Gradient elution with optimized flowrate sections leads to balanced numbers of precursors eluting at the same time and maximized MS utilization time.

#### 3.5.1 NanoLC setup 1

We achieved the highest number of identifications with a 60-min gradient elution. Our optimized workflow is based on a 37-min active elution gradient and a “60 + 1”-minute run-to-run time allowing the analysis of 24 samples per day.

The dead time between the injection and when the first peptides showing up was 19 min with a 300 nL/min flowrate (Supplementary Figure S4). Such dead volume is determined by the system volume and the length of the tubing necessary to connect the injection and trapping valves. Although it was possible to optimize the MS utilization by accelerating the elution of hydrophobic peptides, column washing, and equilibration, faster than the 60-min methods on the M-class and NanoAcquity LC setup resulted in drastically reduced MS utilization time and reduced proteome coverage.

By using a very thin autosampler needle and advanced plate generator software, we were able to adapt the autosampler for injections from commercially available low-volume 1536-well PCR plates with a 4 µL total volume per well. Well-to-well distance is 0.25 mm and the limit of spatial resolution of the autosampler needle movements is 0.1 mm. We circumvented this limitation by rounding up or down to 0.1 mm and generating four 384-well plate templates, each working in its sector of a 1,536-well PCR plate. Although the data quality acquired from a 1536-well plate was similar to that of a 384-well plate (Figure 6), it drastically reduced evaporation rates making sample handling, storage, and transportation considerably more convenient.

#### 3.5.2 NanoLC setup 2

**FIGURE 7:**
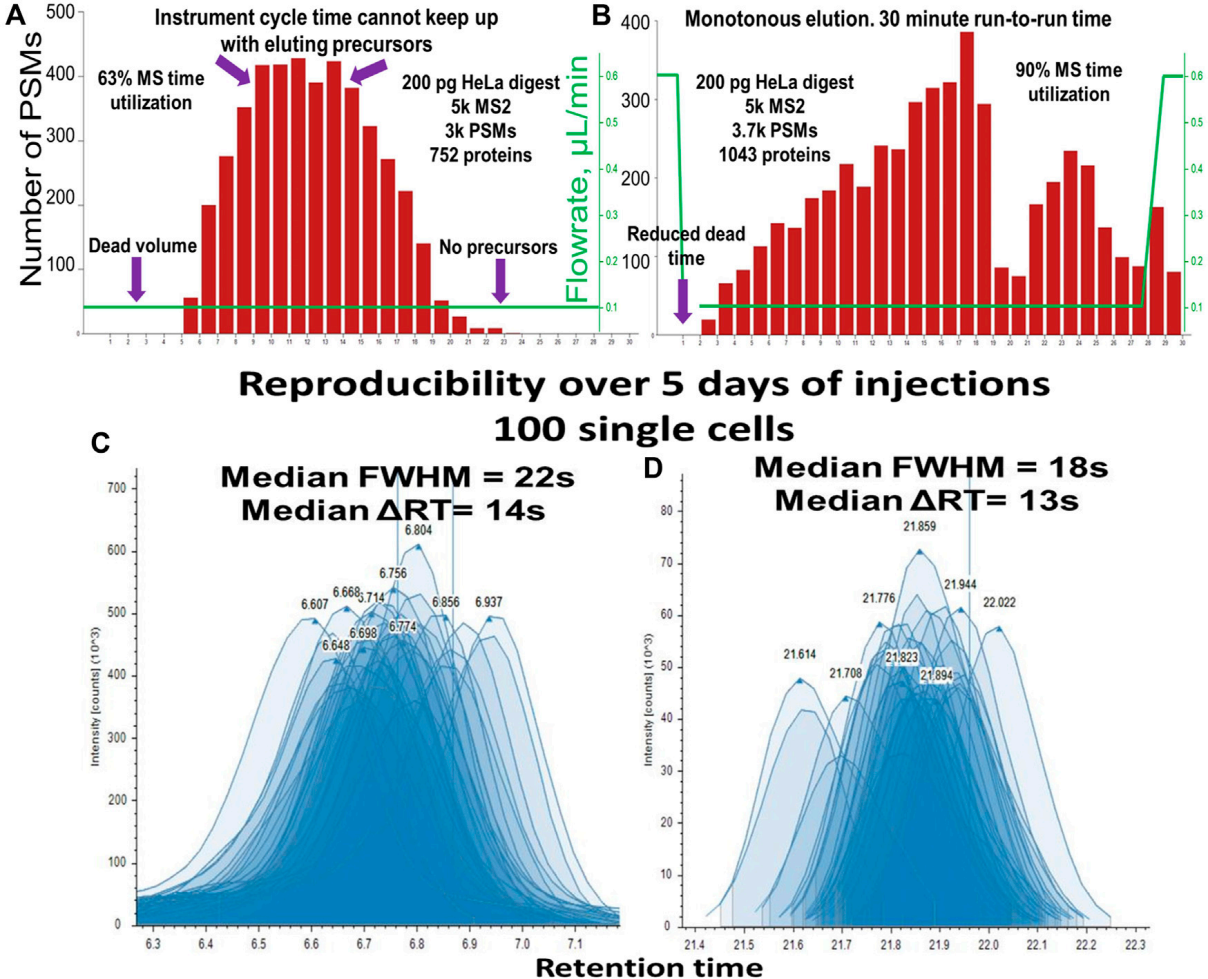
PSM elution profile representing the MS time utilization for “30 + 1” minute run-to-run method (nanoLC setup 2: Vanquish Neo, 50 µm i.d.) for **(A)** Constant flowrate, **(B)** Balanced variable flowrate gradient. Reproducibility of peak shape and RT of 120 injections over 5 days of uninterrupted analysis **(C)** peptide in the first 1/3 of the gradient, median FWHM = 22 s, median ΔRT = 14 s, **(D)** peptide in the last 1/3 of the gradient, median FWHM = 18 s, median ΔRT = 13 s.

**FIGURE 8:**
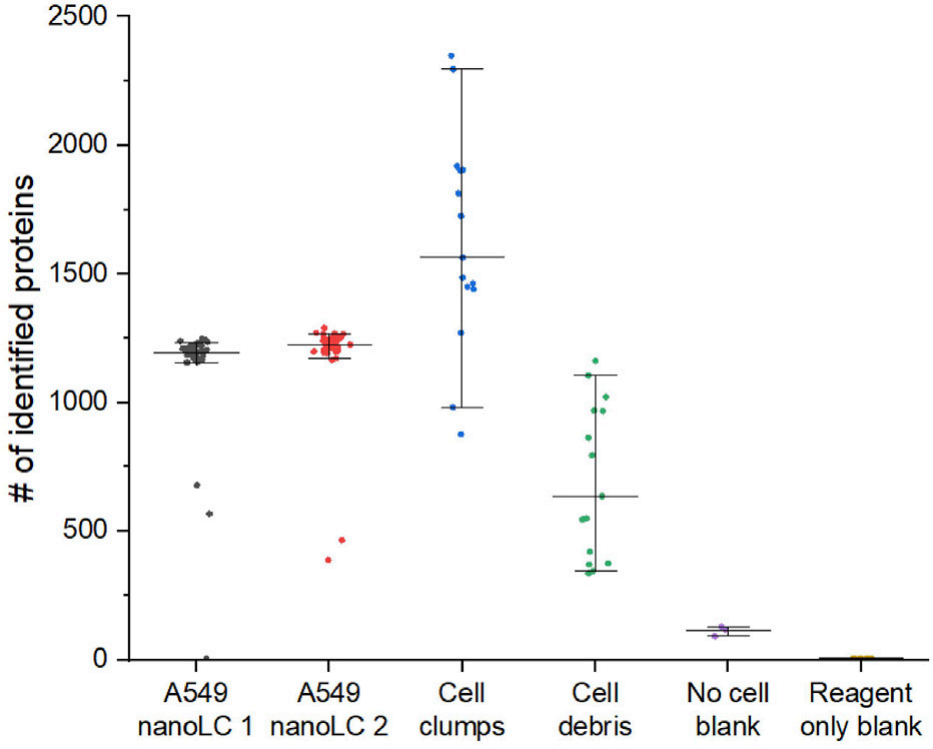
Characterization of D100 dispensing accuracy by proteomics on example of A549 lung adenocarcinoma cells. Grey – 45 samples labeled by D100 as single cells analyzed on nanoLC setup 1. Red – 38 samples labeled by D100 as single cells analyzed on nanoLC 2. Blue – 15 samples labeled by D100 as cell clumps. Green – 15 samples labeled by D100 as cell debris. Purple – 3 “No cell” blank samples prepared by transferring 400 pL of supernatant of a cell suspension (500 cells/µl) centrifuged at 800 g for 5 min. Yellow – 5 “Reagent only” blanks containing all reagents used for analysis.

The low system volume of the Vanquish Neo improved the ratio of MS utilization time to active elution time in conjunction with faster loading, washing, and equilibration. Constant flowrate gradient elution produced a bell-shaped Peptide Spectrum Match–Retention time (PSM-RT) plot (Figure 7A). Under such conditions, in the middle portion of the gradient and when operated in the DDA mode, the instrument was unable to effectively fragment the overwhelming number of co-eluting precursors, while at the beginning and at the end of the gradient, the peak intensity was improved when the flowrate was accelerated. In addition, column washing and equilibration takes a considerable amount of time if performed at the same flowrate as the separation. We evaluated other label-free methods with the throughput of ∼40–50 samples per day (Phlairaharn et al., 2022; Matzinger et al., 2023b) and found that MS utilization time expressed as active elution time/run-to-run time varied from 70% to 85%. Using an optimized balanced gradient with variable flowrate (Figure 7B) resulted in the identification of ∼3-6 PSMs per second in all parts of the gradient. The optimal workflow included ∼29.0 min of active elution with “30 + 1”-minute run-to-run time, resulting in 93.5% MS utilization time. The flowrate was maintained at 120 nL/min flowrate throughout the active peptide elution (Figure 8B) allowing the optimal ionization efficiency for our setup. While these data were obtained using an Orbitrap Fusion Lumos with a 30-Hz 20-mm Orbitrap detector, any instrumental setup may benefit from balancing the peptide elution and optimized MS utilization time. It is also important to mention that 3 min of “dead” time (calculated as (run-to-run time) - (active elution time)) come from 3 major factors: 1) exactly 1-min delay between sample pick up and sample loading, 2) approximately 30-s delay from the tubing connecting the trapping cartridge, and 3) 30-s delay necessary to pass the dead volume of the system and the column. The first two delays can be shortened resulting in close to 100% MS utilization time. The optimized one-column method with >90% MS utilization time brings the sample-per-day (SPD) throughput into the same range as a multi-trap (Webber et al., 2022) and multi-column system (Kreimer et al., 2022).

The “30 + 1” method was developed and tested on the single cell equivalents of tryptic HeLa digest diluted to concentrations 200 to 1 ng/μL (1 μL and 0.2 µL injection volumes respectively). This method also performed well for single-cell samples at first, but after 3 days of single-cell sample injections, we noticed column poisoning. We assumed it was coming from the accumulation of hydrophobic lipids and other small molecules present in higher quantities in single-cell samples compared to diluted HeLa digest. To remediate, we added 1 extra minute of column washing time at the end of the gradient. The resulting “30 + 2” method (90.6% MS time utilization) showed excellent peak shape and RT reproducibility across hundreds of injections performed over multiple days (Figures 7C, D).

Both nanoLC setups combined with the MS acquisition parameters described below showed similar proteome coverage (Figure 5). Unfortunately, at this time, the NanoLC setup 2 system could not be configured to work with 1536-well plates.

#### 3.5.3 MS acquisition

We thoroughly optimized the MS acquisition parameters for low-number-of-cells and single-cell proteomic samples (Supplementary Table S2). Compared to bulk samples, much higher automated gain control target values (AGC) and ion injection times were necessary to boost the instrument sensitivity. To have a reasonable number of scans per peak without compromising MS2 acquisition (Supplementary Table S3), the cycle time was optimized in the range of 1–3− s, with an optimal value of 1.7 s. Given that the median FWHM for chromatographic peak in “30 + 2” method described above was 23 s, it resulted in 10–15 MS1 scans covering every peak providing a reliable and reproducible quantification for the majority of precursors.

AGC was set at 200% and 250% for MS1 and MS2 events, respectively. Ion injection time was optimized for MS1 and MS2 scans in the range from 50 to 400 ms. The optimal values of 118 ms (resolution 120 k) and 246 ms (resolution 60 k) were used for MS1 and MS2 events, respectively. The optimized quadrupole isolation window width was either 1.6 *m/z* for regular DDA or 12 *m/z* for WWA (Mayer et al., 2022).

As seen in Figure 4, the majority of peptide-like features found in a single-cell sample exhibited low abundance (based on peak intensity), with abundances ranging from 10^3^ to 10^7^. Contaminant proteins produce <1% of peaks with abundances 10^8^–10^9^. Based on these values, we determined that the optimal intensity threshold to trigger MS2 events was 3e3. Despite the low abundance, very high AGC value and injection time enabled accumulation of sufficient ion packages that resulted in reasonable quality fragmentation spectra (Supplementary Figure S5).

Although these parameters were optimized for our setup, newer, faster, and more sensitive systems may decrease the amount of injection time devoted to every MS1 and MS2 event (Heil et al., 2023).

### 3.6 Data acquisition strategy and search engine comparison

We next evaluated whether processing the data using different software and search engines would alter the results. We found minor differences when comparing the results obtained using Andromeda and Sequest HT search engines (Supplementary Figure S6A). Upon closer examination, it became clear that in the small fraction of differential proteins, borderline cases resulted in a different protein annotation due to slight differences in search engine peptide score ranking and parsimony rules applied [data not shown]. Compared to the acquisition with narrow isolation windows, implementation of WWA strategy combined with the Chimerys search engine led to improvements in the proteome coverage.

We also evaluated methods based on data independent acquisition. Library free database search using DIA-NN, FragPipe, and Chimerys search engines resulted in comparable to WWA number of identified proteins for 200 pg HeLa digest (Supplementary Figure S7). Despite the assumed 1% FDR, we found that the overlap between different search engines for DIA data from the single-cell samples was poor (Supplementary Figure S6B) and there were discrepancies in the annotation of the very same spectra. Similar observations were made previously (Ye et al., 2023; Wang et al., 2022; Zhang et al., 2023; Lou et al., 2023). Our observations let us conclude that WWA was the best acquisition strategy for our instrumentation and targeted throughput.

### 3.7 Single-cell proteomics confirms accuracy of single-cell detection by impedance

For the dataset shown in Figure 8, we analyzed in a randomized order 45 single A549 cell samples on nanoLC 1 and 38 samples using nanoLC 2. Simultaneously with that, we also evaluated wells from multiple plates that were marked as cell debris and cell clumps by the D100 to compare to the single-cell samples.

It can be noticed that the number of proteins identified in a small fraction of samples labeled by the D100 dispenser as single cells looks drastically different from the rest. From this and other datasets, we concluded that the fraction of such samples is 6%–7%, which is in perfect agreement with the results obtained by evaluating the performance of dispensing of single cells by imaging (Table 1). The proteome coverage of the outliers is consistent with that of cell debris and rarely with the very low number of protein counts commonly observed for reagent-only blanks, which makes it easy to filter them out from the dataset. Since none of the proteomics data from cell-debris nor the cell-clump samples were similar to the single-cell samples after filtering, we assumed that none of the single cells were erroneously assigned as cell debris or cell clumps, which led us to conclude that single-cell detection by impedance is highly accurate.

**FIGURE 9:**
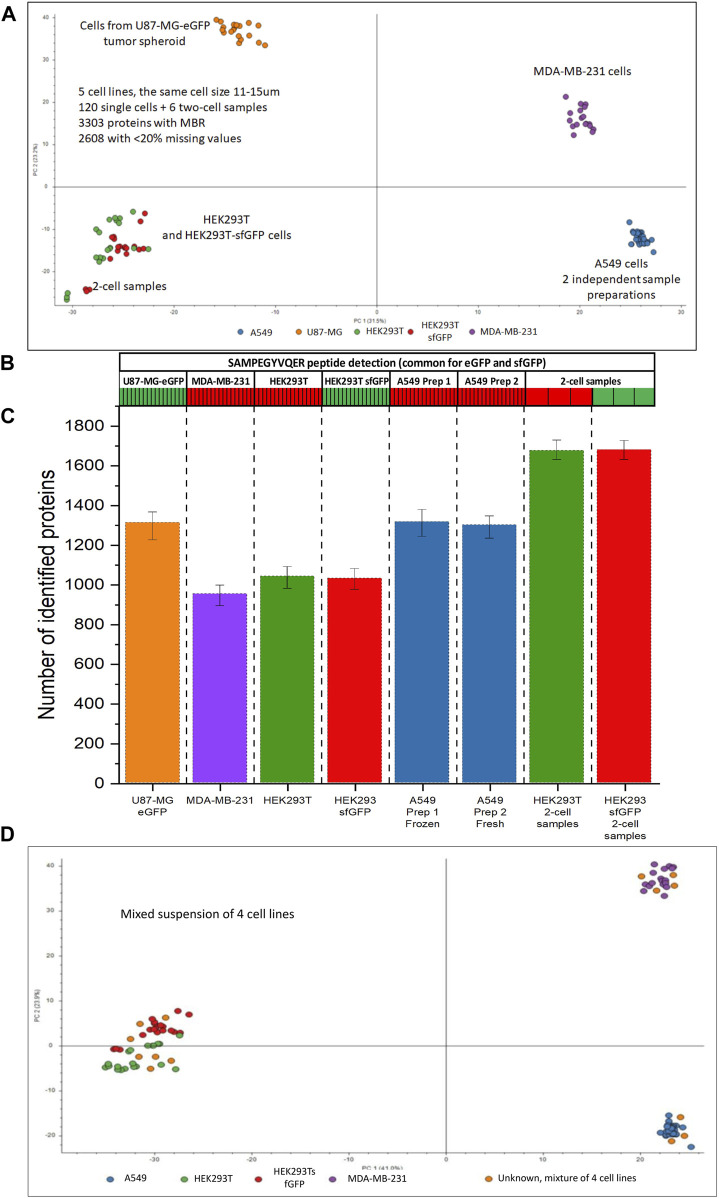
Single-cell analysis of 5 cell lines originating from different tissue types. **(A)** PCA plot for HEK293T (green), HEK293/sfGFP cells (red), A549 (blue), MDA-MB-231 cells (purple), and U87-MG/eGFP (orange), each cell line is represented by 17 single cells, each dot represents a single cell. A549 cell line is represented by 2 batches, 17 cells each, prepared 5 days apart. **(B)** Heatmap showing the detection of a peptide SAMPEGYVQER++ common for sfGFP and eGFP, **(C)** Numbers of proteins identified without MBR in 5 cell lines and 2-cell samples. **(D)** PCA plot for HEK293T (green), HEK293/sfGFP cells (red), two batches of A549 cells (blue), MDA-MB-231 cells (purple), and their mixed-cell suspension (orange dots). Data were acquired using NanoLC setup 2.

Figure 9D shows that 17 cells from the mixed-cell suspension of 4 cell lines landed in the clusters corresponding to the known cell types. The distribution of the dispensed cell types seems to be random, for approximately equal number of the cells from the mixed-cell suspension can be assigned to each of the clusters.

### 3.8 Demonstration of the robustness of developed single cell proteomics pipeline

We applied the best workflows for NanoLC setups 1 and 2 to 6 cell lines having similar cell sizes 11–15 µm and originating from different tissue types: HEK293A, HEK293-sfGFP, HEK293-eGFP, MDA-MB-231, A549, and U87-MG-GFP tumor spheroids.

The study was designed to stress test the important attributes of robustness of our workflows:

1) Ability to differentiate cell phenotypes was tested on A549 lung cancer cells, MDA-MB-231 breast cancer cells, and U87-MG-eGFP brain cancer cells;
2) Applicability of the workflow to ultra-low numbers of cells, a few 2-cell samples, was tested;
3) Dispensing and processing of cells form a mixed-cell suspension containing 4 cell types (HEK293T, HEK293/sfGFP cells, A549, and MDA-MB-231 cells);
4) Cells from dissociated U87-MG-GFP tumor spheroids were used to simulate future experiments with organoids and tissue samples;
5) One batch of processed and trypsin-digested A549 cells was frozen at −80°C for 5 days before the analysis;
6) Detectability of engineered proteins in single cells using eGFP and sfGFP as examples;
7) Ability to differentiate very similar cells types: HEK293T and HEK293sfGFP are the same cell line before and after the genetic manipulation.

As can be seen on Figure 9A, different cell types can be easily differentiated using PCA. 950–1,300 proteins were identified in 5 cell lines. 17 cells of each cell type were analyzed. With MBR algorithm ≈3,300 proteins were identified in the whole dataset, ≈2,600 of them had less than 20% missing values in at least one cell line. In spite of the assumption that different tissue types would have many unique proteins, a large portion of the proteome, ≈1930 had less than 20% missing values across all cell lines. Abundances of these proteins were used for statistical analysis.

Approximately 1,600 proteins (No MBR) were identified in 2-cell samples (Figure 9C). Although 2-cell samples can be clearly told apart, they co-cluster with the corresponding single-cell samples of the same type and are very distant from the clusters corresponding to the other cell types (Figure 9A). This observation allowed us to conclude that the cluster separation was driven by the proteome profile rather than by the total amount of protein injected with the cell.

We did two sample preparations of A549 cells to evaluate the ability to store and transport the samples. Two different 10-cm cell culture dishes with A549 cells at 90% confluency were harvested 5 days apart. 384-well plate with freshly prepared single-cell samples from the first dish was stored at −80°C and thawed at room temperature after the cells from the second dish were prepared in the second 384-well plate. After that, the samples from two sample preparations were analyzed in a randomized order in the same batch with the other cells represented on Figure 9. The average number of proteins identified in the cells prepared on different days was similar: 1,284 and 1,271 proteins, respectively. The frozen and later thawed single-cell samples completely overlapped with the freshly prepared single-cell samples on the PCA plot (Figure 9A).

### 3.9 Evaluating the outcomes of genetic manipulations and detecting engineered proteins in individual cells

**FIGURE 10:**
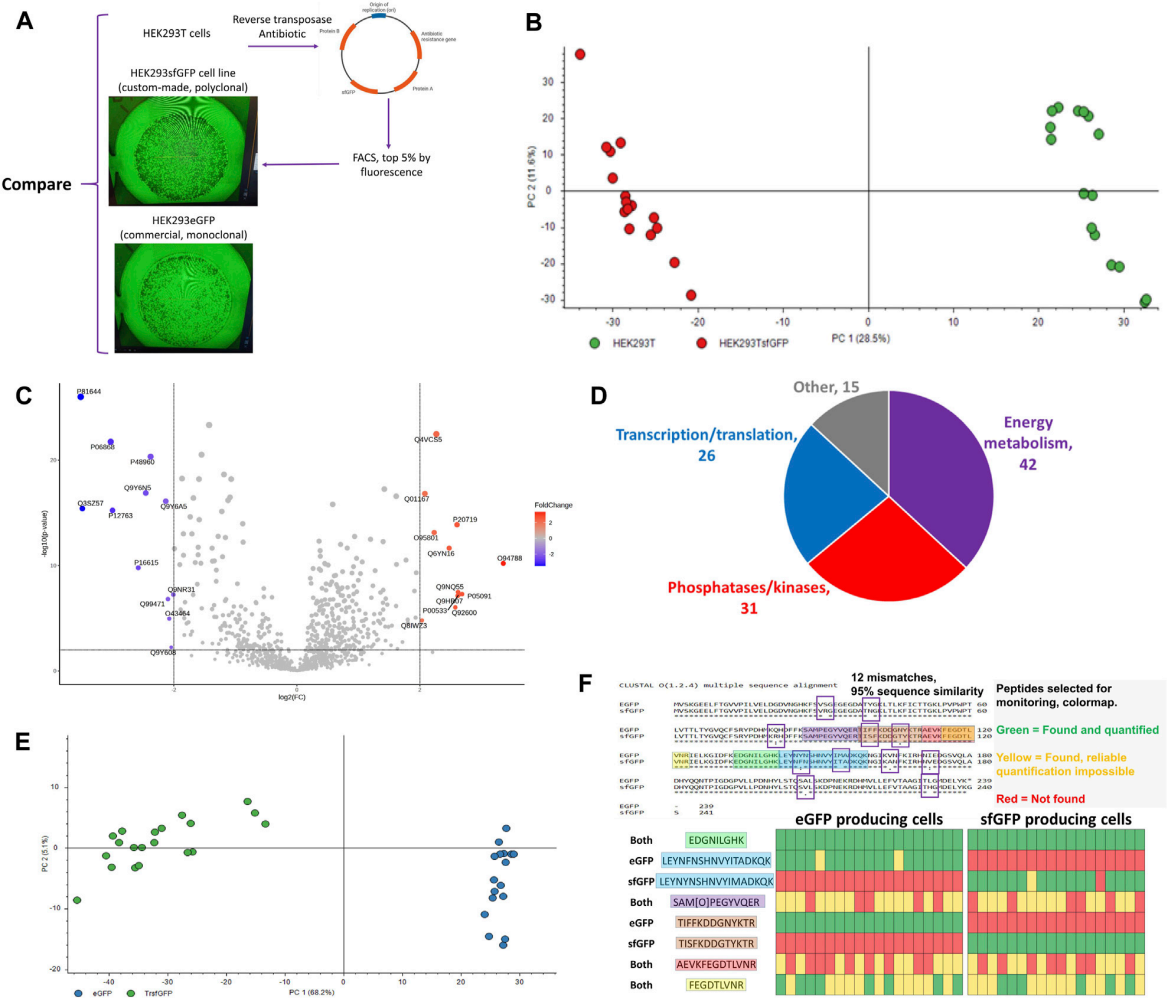
**(A)** HEK293-derived cell lines used in the study, **(B)** PCA plot for the proteomic profiles of parental HEK293T (green) and descendant genetically modified cell line HEK293sfGFP (red), each dot represents an individual cell, **(C)** Volcano plot highlighting the differential proteins between HEK293T and HEK293sfGFP (log2(FC) = 2, *p* < 0.01), **(D)** Functional distribution of differential proteins, **(E)** Principal component analysis of single HEK293/eGFP and HEK293/sfGFP cells, **(F)** eGFP and sfGFP sequence aligned and heatmap for the presence and absence of peptides which are either unique or common for eGFP and sfGFP. The version of Figure 10B with 2-cell samples included can be found in Supplementary Figure S9. The data for Figures 10B–D were produced using NanoLC setup 2 and data resulting in Figures 10E, F were produced using NanoLC setup 1.

The developed workflow was used for the detection of expressed engineered proteins in individual cells. HEK293sfGFP cells are a polyclonal cell line produced from HEK293T cells by incorporating GFP into the cell genome using a reverse transposase (Figure 10A). HEK293GFP is a monoclonal commercially available GFP producing cell line. Although these three cell lines are the descendants of the same parent HEK293 cell line, the single cell proteome coverage was enough to clearly differentiate them using PCA (Figures 10B, E) or a Volcano plot (Figure 10C). GFP was not included in the FASTA database in order to avoid GFP-related bias in the statistical analysis. Nevertheless, presence or absence of unique GFP peptides can be easily used for classification of these three cell lines (Figures 9B, 10F). The collected data show that it is possible to distinguish between stably expressed superfolder green fluorescent protein (sfGFP), enhanced GFP (eGFP), and transiently transfected sfGFP at the single-cell level. Sequence overlap between the sequences of proteins in this study was >98% (Figure 10F). Differential tryptic peptides corresponding to the same part of the protein sequence were different in only one or two amino acids. Due to high sequence similarity, such tasks cannot be approached by competing techniques such as mass cytometry and flow cytometry.

Proteins affected by the genetic manipulation, 58 upregulated and 56 downregulated proteins (*p* < 0.01, log2FC > 2), can be divided into 3 groups (Figure 10D) by the biochemical processes they are involved in: 1) nucleotide and nucleic acid processing enzymes, 2) phosphatases/kinases, 3) energy metabolism. Such changes are consistent with the nature of the genetic transformation involving the overtranscription and overtranslation of the bioengineered proteins. Figure 10E emphasizes the protein content differences in the eGFP and sfGFP cell lines resulting in separation of the cell-types in the PCA plot. The cell identity can be unambiguously stated based on the presence or absence of unique differential peptides in sfGFP (Figure 10F).

## 4 Conclusion

In this study, we adapted a low-cost TIJ dispensing platform for dispensing of single cells and processing of single cells for MS proteomics. We investigated reagent and single cell dispensing accuracy. Our results demonstrated that TIJ dispenses up to 100 intact, viable cells per minute, which can be used for single cell mass spectrometry and other applications, such as single cell cloning.

The work focused on using exclusively commercially available components and making the workflow robust and accessible to other research groups. The developed workflow enables preparation of up to 15,000 single-cell samples per day. We achieved label-free identification of 1,000–1,300 proteins on average from a single cell per run for multiple cell types and more than 3,000 proteins with Match-Between-Runs algorithm. Our separation methods enable high MS utilization time usage, allowing similar throughput levels to multicolumn systems. The throughput of these methods ranges from 24 to 96 samples per day, depending on the LC system used. Further increase in throughput is achievable with faster instrumentation or by sacrificing proteome depth.

We developed an accessible, versatile, and automated novel sample preparation workflow which can be easily adopted by other research groups interested in single-cell proteomics. Accessibility of our methods was confirmed in a short study performed in another laboratory (Sanchez-Avila et al., 2023). The developed one-pot sample preparation method is compatible with commercially available 96-, 384-, 1536-well plates, and LC vials. The microplates with the samples were directly placed into the LC autosampler, and multiple nanoLC setups with commercially available columns were tested. The TIJ-enabled workflow was used for the analysis of individual cells from different cell lines and tumor spheroids, for monitoring the consequences of genetic manipulations at the single-cell level, and for trace analysis and screening of individual analytes—bioengineered proteins—in individual cells. MS-based single-cell proteomics provides a unique capability to track highly homologous engineered proteins while simultaneously acquiring the whole single-cell proteomic profile.

To conclude, TIJ dispensing was advantageous for both handling of very low reagent volumes (pico-, nano-, microliter range) and for fast single-cell dispensing. The gain in performance and proteome coverage was achieved due to the careful consideration of multiple factors resulting in optimized sample preparation handled by the TIJ device and thoroughly optimized data acquisition strategies. The wide availability of all supplies, rapid cell and liquid handling, compatibility with common nanoLC systems, and reasonable throughput make our workflow reproducible, accessible, and transferable to other laboratories.

## Data Availability

The datasets presented in this study can be found in online repositories. The names of the repository/repositories and accession number(s) can be found in the article/Supplementary Material.
