# Supplementary material for: Thermal inkjet makes label-free single-cell proteomics accessible and easy
Source: Front Chem. 2024 Aug 21;12:1428547. doi: 10.3389/fchem.2024.1428547 (PMC11371764; doi:10.3389/fchem.2024.1428547)
Supplement: Supplementary file 1 [file DataSheet1.docx]

**Supplementary Materials**

**Thermal inkjet makes label-free single-cell proteomics accessible and easy**

Stanislau Stanisheuski^1^, Arpa Ebrahimi^1^, Kavi Aashish Vaidya^2^, Hyo Sang Jang^3^, Liping Yang^1^, Alex Eddins^2^, Carrie Marean-Reardon^2^, Maria Clara Franco^4,5^, Claudia Susanne Maier^1*^

^1^Department of Chemistry, Oregon State University

^2^Department of Biochemistry and Biophysics, Oregon State University

^3^HP Inc.

^4^Center for Translational Science, Florida International University

^5^Department of Cellular and Molecular Medicine, Herbert Wertheim College of Medicine, Florida International University

Supplementary Materials

Table of Contents

Figure S1. U87-MG-GFP tumor spheroids.

Figure S2. Dispensing of MassPREP standard by D100.

Figure S3. 1/3 cell PCA (MDA-MB-231 vs HEK293A)

Figure S4. PSM distribution for 60-minute gradient on M-class Acquity UPLC

Figure S5. Example of a fragmentation spectrum for a very low-abundant peptide

Figure S6. Comparison of search engines for DDA and DIA.

Figure S7. Comparison of proteome coverage by different acquisition strategies

Figure S8. PCA plot of HEK293sfGFPvsHEK293T with two-cell samples.

Table S1. Optimized sample preparation methods

Table S2. Optimization of MS acquisition

Table S3. Optimized MS methods

Table S4 and S5. LC methods

Appendix 1. Consumables


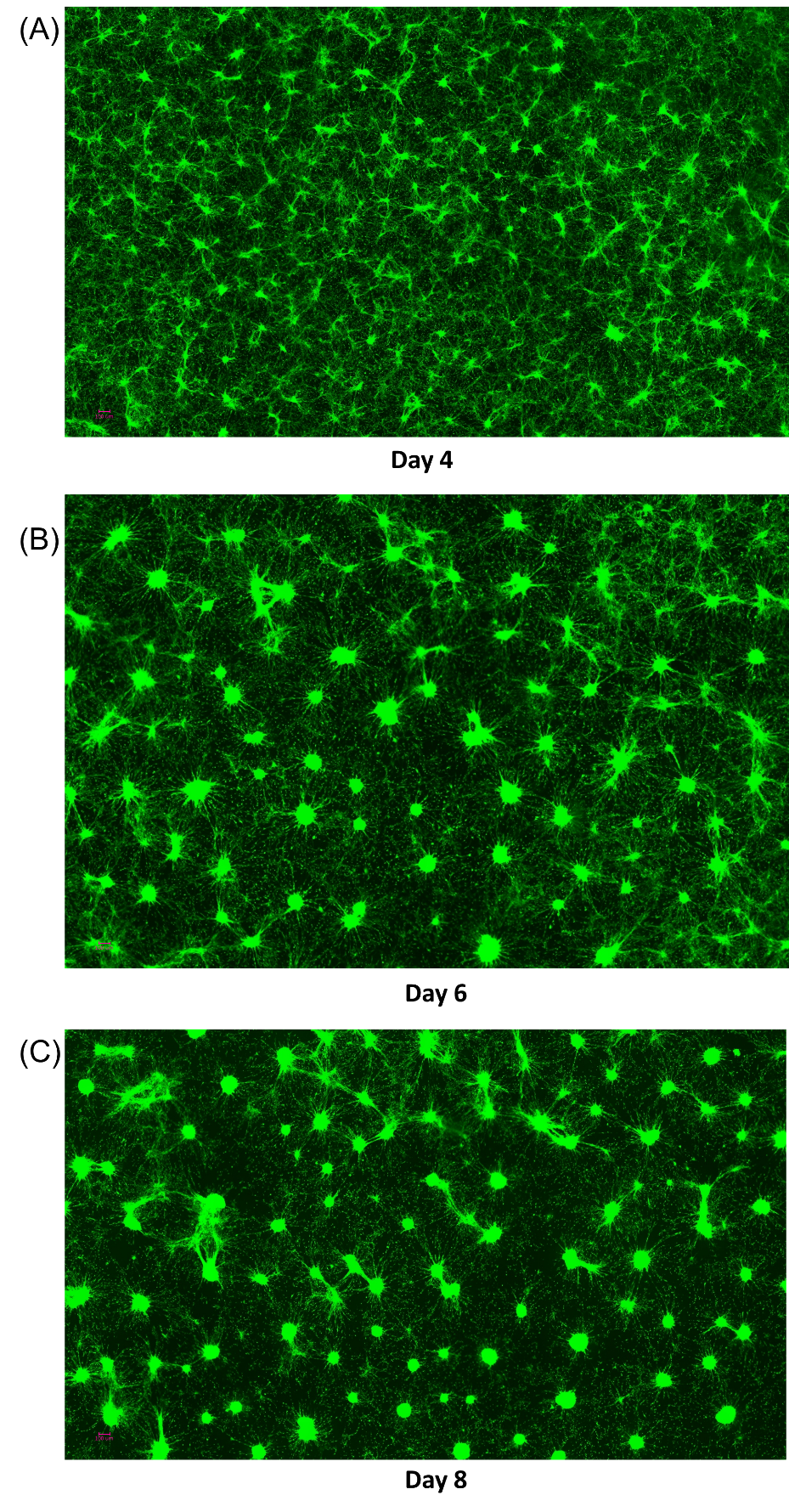


**Figure S1** – U87-MG-GFP tumor spheroids after a) 4 days, b) 6 days, and c) 7 days of growth


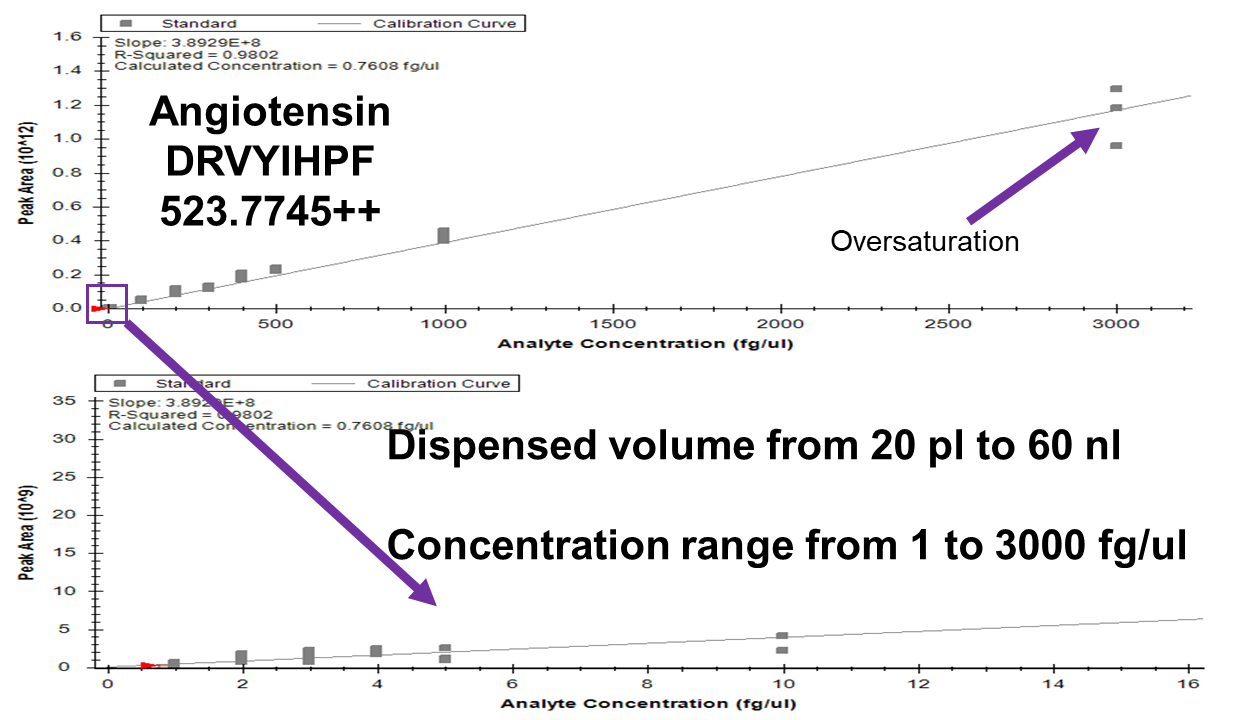


**Figure S2** – dispensing of MassPREP peptide standard. PRM of DRVYIHPF peptide 523.7745++ from angiotensin.


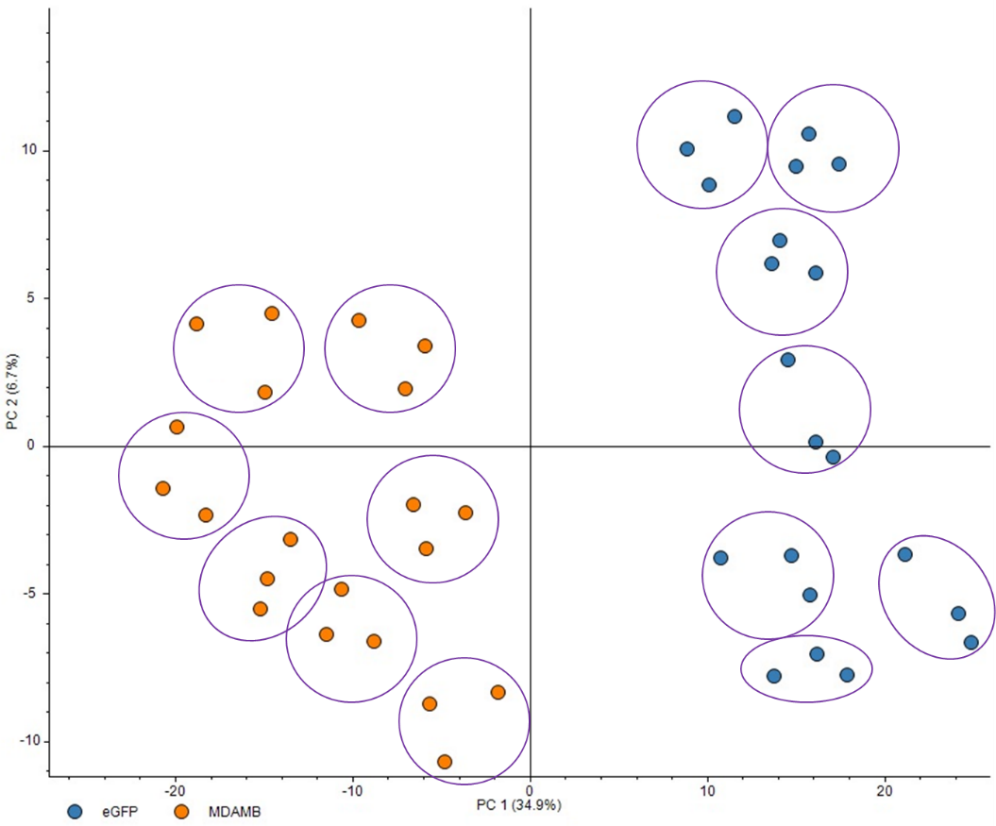


**Figure S3** – PCA of ≈1/3 of single MDA-MB-231 and HEK293/eGFP cell. Each purple bubble indicates three 1 µL injections from the same vial with a single cell digest prepared in 4 µL volume.


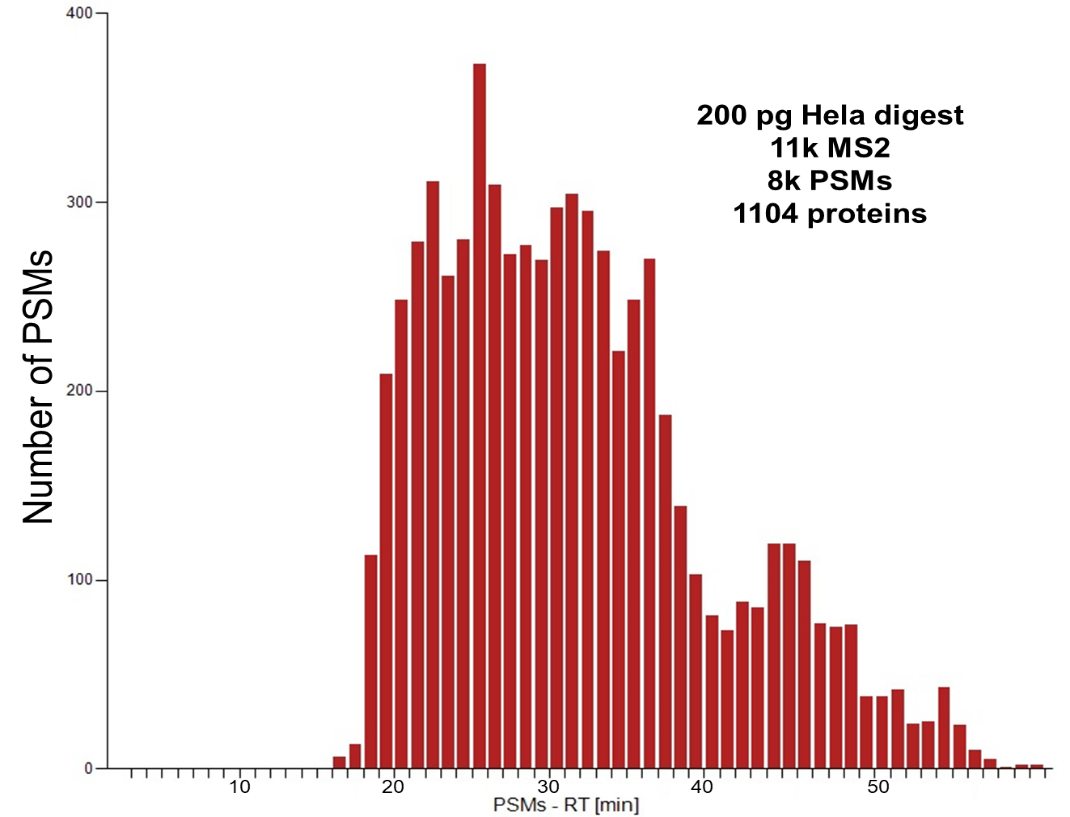


**Figure S4** – PSM distribution for 60-minute gradient at 300 nL/min using an Acquity UPLC M-Class system


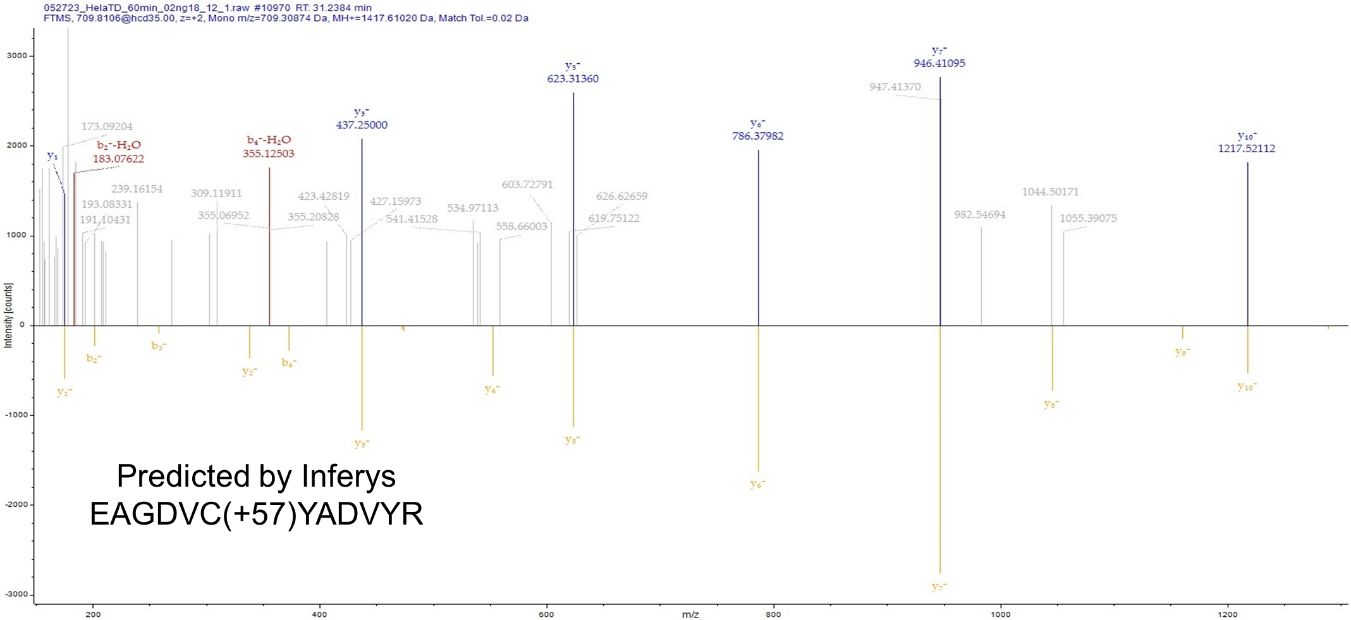


**Figure S5 –** a) example of an annotated MS/MS spectrum of very low abundance identified as EAGDVC(+57)YADVYR (709.811++) in 200 pg/µL HeLa digest sample, b) MS/MS spectrum for EAGDVC(+57)YADVYR++ peptide predicted by the Inferys algorithm.


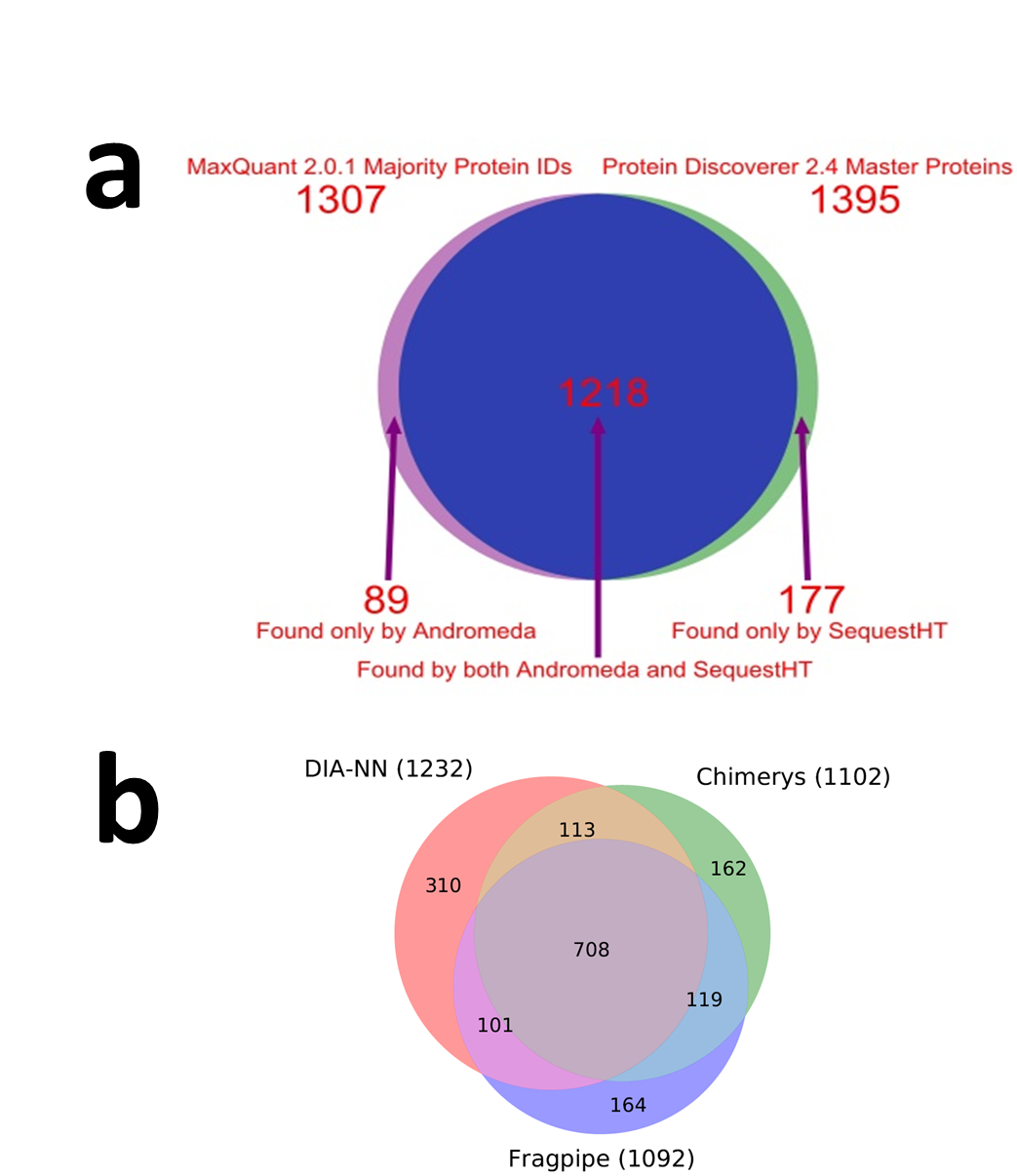


**Figure S6** – comparison of protein IDs identified by a) Andromeda and SequestHT search engines in 20 individual HEK293T cells analyzed using DDA, b) DIA-NN, Chimerys, and Fragpipe search engines in a single HEK293T cell analyzed by DIA.


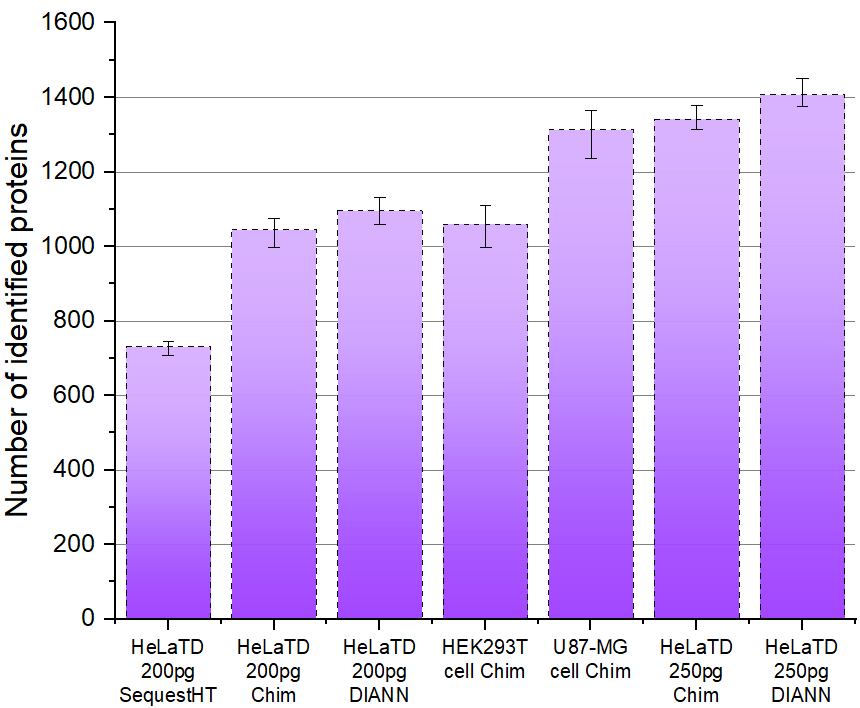


**Figure S7** – comparison of protein IDs identified by DDA (Sequst HT), WWA (Chimerys), and DIA (DIA-NN) strategies in 200 and 250 pg of HeLa digest and in single HEK293T and U87-MG-GFP cells


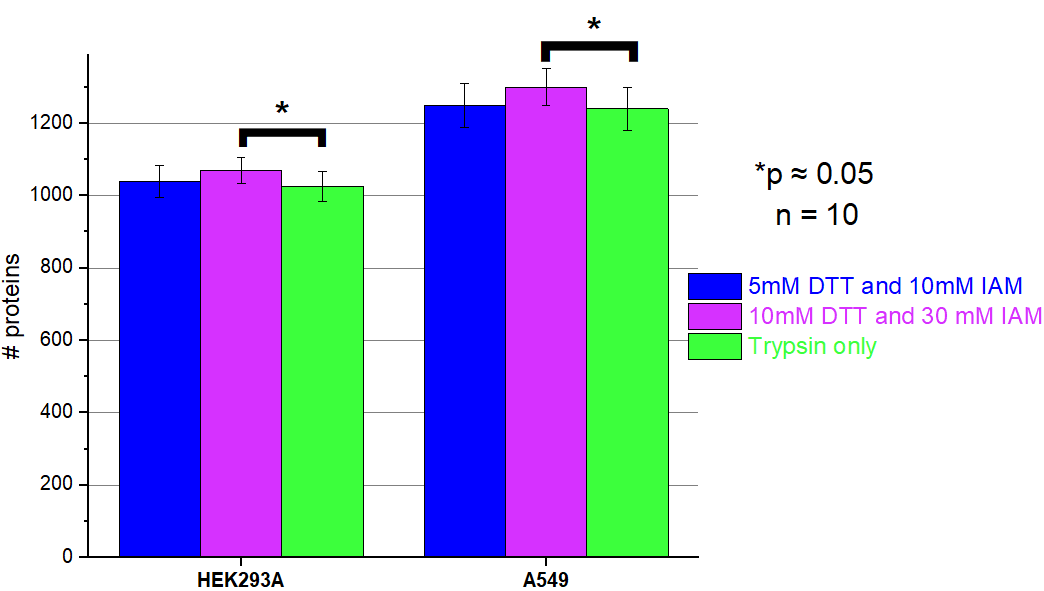


**Figure S8** – number of identified protein in individual HEK293A and A549 cells processed with and without reduction and alkylation steps, each bar represents 10 cells.


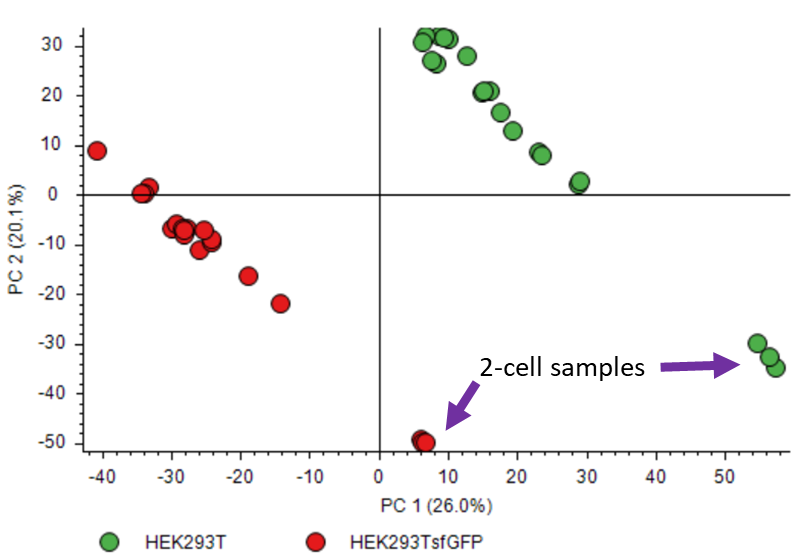


**Figure S9** – PCA plot for the proteomic profiles of parental HEK293T (green) and descendant genetically modified cell line HEK293sfGFP (red), each dot represents individual cell, except for the samples labeled as 2-cell samples.

**Table S1.** Optimized sample preparation methods

| Step | Methods for trape-and-elute | | Method compatible with direct injection on analytical column |
| --- | --- | --- | --- |
|  | No alkylation | Reduction+Alkylation | Reduction+Alkylation |
| 1 | Dispense 1.2 µL mixture of 5ng/ µL trypsin (Rapid) in 50 mM PBS and centrifuge. | Dispense 1.2 µL mixture of 5ng/ µL trypsin (Rapid), 10 mM DTT in 50 mM PBS and centrifuge. | Dispense 1.2 µL mixture of 5ng/ul trypsin (Rapid), 10 mM DTT, 0.02% Rapigest, 0.07% DDM in 100 mM TEAB and centrifuge. |
| 2 | Dispense single cells, seal, and centrifuge | Dispense single cells, seal, and centrifuge | Dispense single cells, seal, and centrifuge |
| 3 | Incubate for 25 minutes at 75°C | Incubate for 25 minutes at 75°C | Incubate for 25 minutes at 75°C |
| 4 | Dispense 1.2 µL of 5 ng/µL trypsin in 50 mM PBS, seal, and centrifuge | Dispense 1.2 µL of 5 ng/µL trypsin, 30 mM IAM in 50 mM PBS, seal, and centrifuge | Dispense 1.2 µL of 5 ng/µL trypsin, 30 mM IAM in 100 mM TEAB, seal, and centrifuge |
| 5 | Incubate for 25 minutes at 75°C | Incubate for 25 minutes at 75°C | Incubate for 25 minutes at 75°C |
| 6 | Place the plate into the autosampler and inject 2 µL | Place the plate into the autosampler and inject 2 µL | Place the plate into the autosampler and inject 2 µL |
|  | Centrifuge in these protocols means a 2-minute spin at 2200xg but acceleration and breaking take a full minute for the plates. | | |
|  | Optionally, samples can be frozen at -80°C for at least 2 months and/or transported on dry ice after step 5 | | |

**Table S2.** Parameter ranges selected for optimization for MS acquisition

| **Parameter optimization range** | Method 1 | Method 2 |
| --- | --- | --- |
| **Global settings** |  |  |
| Spray voltage, V | 2000-2500 | 2000-2500 |
| Ion transfer tube temperature, °C | 150-300 | 150-300 |
| MS1 events |  |  |
| Resolution MS1 | 60000-240000 | 120000 |
| Scan Range lower limit, Th | 300-400 | 300-400 |
| Scan Range upper limit, Th | 900-1575 | 900-1575 |
| AGC target, % | 150-350 | 150-350 |
| Maximum injection time, ms | 54-400 | 54-400 |
| S-lens RF, % | 30-60 | 50 |
| Cycle time, s | 1-4 | 1-4 |
| **MS2 events** |  |  |
| Resolution MS2 | 15000-240000 | 30000-120000 |
| AGC target, % | 50-400 | 50-400 |
| Maximum injection time, ms | 54-400 | 54-400 |
| Precursor intensity threshold | 1000-20000 | 3000-10000 |
| Isolation window, Th | 0.4-2.5 | 6-16 |

**Table S3.** Optimized DDA and DDA-WWA methods

| **Settings** | Method 1 | Method 2 |
| --- | --- | --- |
| **Global settings** |  |  |
| Spray voltage, V | 2300 | 2300 |
| Ion transfer tube temperature, °C | 200 | 200 |
| **MS1 events** |  |  |
| Resoltuion MS1 | 120000 | 120000 |
| Scan Range, Th | 375-1575 | 375-900 |
| AGC target, % | 250 | 250 |
| Maximum injection time, ms | 118 | 118 |
| S-lens RF, % | 50 | 50 |
| Cycle time, s | 3 | 1.7 |
| **MS2 events** |  |  |
| Resolution MS2 | 60000 | 60000 |
| Scan Range First mass, Th | 100 | 100 |
| AGC target, % | 200 | 200 |
| Maximum injection time, ms | 246 | 246 |
| Precursor intensity threshold | 5000 | 3000 |
| Precursor charge state | 2-5 | 2-5 |
| Dynamic exclusion time, s | 30 | 30 |
| Isolation window, Th | 1.6 | 12 |

**Table S4.** Parameters used for NanoAcquity and M-class UPLC

| Trapping method |  |  |
| --- | --- | --- |
| Trapping time | 1.5 min |  |
| Flowrate | 5 µL/min |  |
| Solvent composition | 3% ACN |  |
| Advanced parameters | Desired flow rate is applied immediately |  |
|  |  |  |
| Analytical method |  |  |
| Run time | 60 min |  |
| Actual run-to-run time | 62 min |  |
| Advanced parameters |  |  |
| Air gaps, pre- and post-aspirate | 0.5 µL |  |
| Needle placement (from bottom) | 0.1 mm |  |
|  |  |  |
| Time, min | % ACN | Flowrate, nL/min |
| 0 | 3 | 300 |
| 3 | 10 | 300 |
| 52 | 30 | 300 |
| 54 | 90 | 300 |
| 56 | 90 | 300 |
| 57 | 3 | 300 |
| 69 | 3 | 300 |

**Table S5.** Parameters used for Neo Vanquish

| Run time | 30 min |  |
| --- | --- | --- |
| Actual run-to-run time | 32 min |  |
| LoadingVolume | 1.5 µL |  |
| Loading flow rate | 5 µL/min |  |
| Max loading pressure | 11603 psi |  |
| BottomLift | 0.1 mm |  |
| TrapColumnFlushDirection | Backward |  |
| No air gaps |  |  |
| SolventForTrapping | 3% ACN |  |
|  |  |  |
| Time, min | % ACN | Flowrate, nL/min |
| 0.0 | 3 | 600 |
| 2.0 | 10 | 600 |
| 3.0 | 10 | 120 |
| 25.0 | 30 | 120 |
| 27.0 | 95 | 120 |
| 29.5 | 95 | 600 |
| 30.0 | 3 | 600 |
| No column equilibration, Volume set to 0 |  |  |

**Appendix 1. Consumables**

**384-well plates:**

Thermo 60180-P139B

https://www.thermofisher.com/order/catalog/product/60180-P139B

Thermo AB2384

https://www.thermofisher.com/order/catalog/product/AB2384

**Columns for Vanquish Neo**

50 um x 15 cm, 2um 100Å

Thermo 164943

https://www.thermofisher.com/order/catalog/product/164943

PepMap™ Neo Nano Trap Cartridge

300 um x 5 mm

https://www.thermofisher.com/order/catalog/product/174500

**Columns for Waters UPLCs:**

ACQUITY UPLC M-Class Peptide BEH C18 Column, 130Å, 1.7 µm, 100 µm X 100 mm, 1/pk

ACQUITY UPLC M-Class Peptide BEH C18 Column, 130Å, 1.7 µm, 75 µm X 100 mm, 1/pk, 186007481

https://www.waters.com/nextgen/us/en/shop/columns/186007481-acquity-uplc-m-class-peptide-beh-c18-column-130a-17--m-75--m-x-1.html

nanoEase M/Z Symmetry C18 Trap Column, 100A, 5 µm, 180 µm x 20mm, 1/pk, 186008821

https://www.waters.com/nextgen/us/en/shop/columns/186008821-nanoease-m-z-symmetry-c18-trap-column-100a-5--m-180--m-x-20mm-1-.html
